# Upregulation of sex-determining region Y-box 9 (SOX9) promotes cell proliferation and tumorigenicity in esophageal squamous cell carcinoma

**DOI:** 10.18632/oncotarget.5160

**Published:** 2015-09-04

**Authors:** Yingcai Hong, Wen Chen, Xiaojun Du, Huiwen Ning, Huaisheng Chen, Ruiqing Shi, Shaolin Lin, Rongyu Xu, Jinrong Zhu, Shu Wu, Haiyu Zhou

**Affiliations:** ^1^ Department of Thoracic Surgery, Shenzhen People's Hospital, the Second Clinical Medical College of Jinan University, Shenzhen 510000, China; ^2^ Department of Traditional Chinese Medicine, The Second Affiliated Hospital of Fujian Medical University, Quanzhou 362000, China; ^3^ Department of Thoracic Surgery, Guangdong General Hospital & Guangdong Academy of Medical Sciences, Southern Medical University, Guangzhou 510080, China; ^4^ Department of Anorectal Surgery, Shenzhen Second People's Hospital, Shenzhen 518035, China; ^5^ Intensive Care Unit, Shenzhen People's Hospital, the Second Clinical Medical College of Jinan University, Shenzhen 510000, China; ^6^ Department of Thoracic Surgery, Quanzhou First Hospital, the Affiliated Hospital of Fujian Medical University, Quanzhou 362000, China; ^7^ Department of Biochemistry, Zhongshan School of Medicine, Sun Yat-sen University, Guangzhou, Guangdong 510080, China; ^8^ State Key Laboratory of Oncology in Southern China, Department of Experimental Research, Sun Yat-sen University Cancer Center, Guangzhou 510060, China

**Keywords:** SOX9, ESCC, proliferation, Akt

## Abstract

Sex-determining region Y-box 9 (SOX9), a vital transcription factor, play important roles in numerous biological and pathological processes. However, the clinical significance and biological role of SOX9 expression has not been characterized in human esophageal squamous cell cancer (ESCC). Herein, we found that SOX9 was markedly upregulated, at both mRNA and protein level, in ESCC cell lines and ESCC tissues and that SOX9 expression was significantly correlated with tumor clinical stage, T classification, N classification, M classification, pathological differentiation, and shorter overall survival. The proliferation and tumorigenicity of ESCC cells were dramatically induced by SOX9 overexpression but were inhibited by SOX9 knockdown both *in vitro* and *in vivo*. Moreover, we demonstrated that upregulation of SOX9 increased the expression of phosphorylated Akt, the cyclin-dependent kinase (CDK) regulator cyclin D1, phosphorylated forkhead box O (FOXO)1, and phosphorylated FOXO3, but SOX9 downregulation decreased their expression, whereas the levels of the CDK inhibitors p21^Cip1^ and p27^Kip1^ were attenuated in SOX9-transduced cells. Taken together, our results suggest that SOX9 plays an important role in promoting the proliferation and tumorigenesis of ESCC and may represent a novel prognostic marker for the disease.

## INTRODUCTION

Esophageal cancer is the eighth most frequently diagnosed cancer and the sixth most common cause of cancer-related death worldwide [1]. Esophageal squamous cell carcinoma (ESCC), which comprises 60–70% of all cases of esophageal cancer worldwide, is more common in the developing world, especially in China, where cases account for about 70% of global occurrence [2–4]. The prognosis of ESCC is quite poor and the overall five-year survival rate is approximately 15%, and most patients die within the first year of diagnosis [5]. Therefore, there is a critically urgent need to identify effective diagnostic markers and targets for esophageal cancer.

Sex-determining region Y-box 9 (SOX9), a member of the SOX family of transcription factors, contains high mobility group box DNA-binding domains. It has been reported that SOX9 plays essential roles in normal embryogenesis, sterol cell differentiation, chondrogenesis, neural crest development and differentiation, as well as in the development of specific cell types and lineages within the central nervous system, pancreas, prostate, intestine, skin, pituitary, heart, kidney, and sensory systems [6–8]. Mutation or abnormal expression of the *SOX9* gene contributes to many diseases, such as campomelic dysplasia and autosomal sex reversal [9–11]. SOX9 is overexpressed in a variety of malignancies and is associated with cancer development or progression. For example, SOX9 is increased in relapsed hormone-refractory prostate cancer, and overexpression of SOX9 enhanced the growth, angiogenesis, and invasion of prostate cancer cells [12]. Xia *et al*. found that SOX9 is upregulated in pancreatic ductal adenocarcinoma (PDAC) and is associated with distant metastasis and unfavorable prognosis, and that upregulation of SOX9 is significantly relevant to tumor proliferation and could be a prognostic biomarker for PDAC [13]. Capaccione and colleagues demonstrated that SOX9 is overexpressed in lung adenocarcinoma and induces lung cancer cell motility and invasion [14]. Importantly, targeting SOX9 by either small interfering RNAs (siRNAs) or microRNAs significantly reduced tumor cell proliferation and invasion, chondrogenic differentiation in human mesenchymal stem cells, or resistance to targeted therapy for lung adenocarcinoma [14–16]. SOX9 is also overexpressed in ovarian cancer and it could be a possible diagnostic marker of ovarian carcinomas [17]. Collectively, these studies indicate that SOX9 functions as an oncogene and might represent an anti-cancer target. However, the clinical significance and biological role of SOX9 expression in ESCC remain unclear.

In the present study, we reported that SOX9 was overexpressed in ESCC and was significantly correlated with high clinicopathological grade and poor prognosis of ESCC. Furthermore, we demonstrated that overexpression of SOX9 promoted cell proliferation and tumorigenesis in ESCC through activation of the Akt signaling pathway. Taken together, our results indicate that SOX9 might be a potential biomarker of ESCC diagnosis and a potential target for ESCC therapy.

## RESULTS

### SOX9 is overexpressed in ESCC cell lines and ESCC tissues

**Figure 1:**
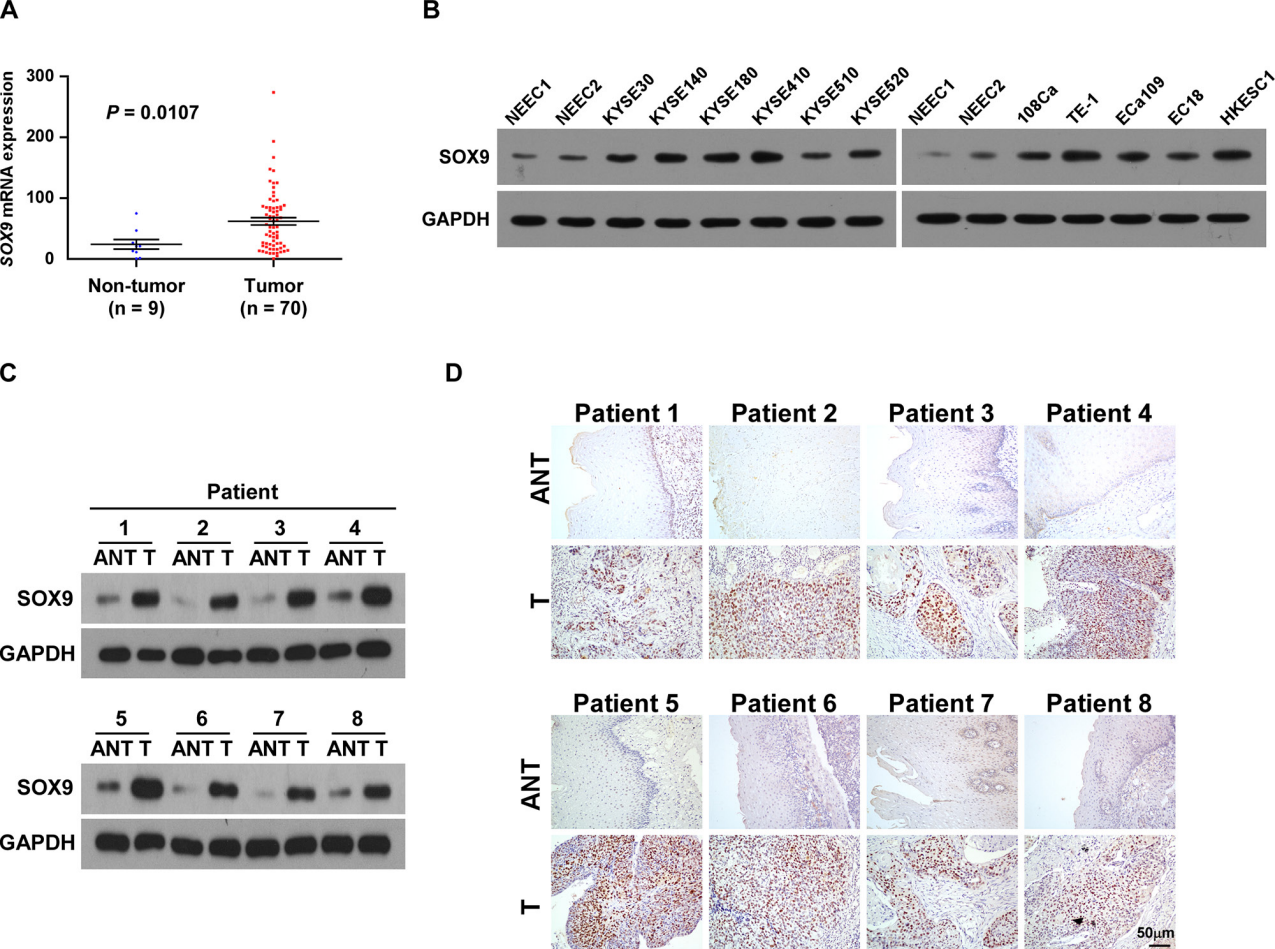
SOX9 is overexpressed in ESCC cell lines and ESCC tissues **A.** *SOX9* is upregulated in ESCC (*P* = 0.0107; TCGA database). Bars represent the mean ± SD of three independent experiments. **B.** Western blotting analysis of SOX9 expression in ESCC cell lines. Glyceraldehyde-3-phosphate dehydrogenase (GAPDH) served as the loading control. NEEC, normal human esophageal epithelial cells. **C.** Western blotting analysis of SOX9 expression in paired ESCC (T) and adjacent non-tumor tissue (ANT) specimens. **D.** IHC examination of SOX9 protein expression in paired ESCC and adjacent non-tumor tissue specimens.

High-throughput assessment analysis using The Cancer Genome Atlas (TCGA) database (http://cancergenome.nih.gov/) revealed that SOX9 was significantly upregulated in ESCC tissues compared with that in non-tumor tissues (non-tumor: *n* = 9, tumor: *n* = 70; *P* = 0.0107; Figure 1A). Consistently, we found that the expression of SOX9, at both protein and mRNA level, was significantly upregulated in all 11 tested ESCC cell lines as compared to the two normal esophageal epithelial cells (NEECs), and in the eight human ESCC samples as compared to the matched adjacent non-tumor tissues (Figure 1B–1D and Supplementary Figure 1A–1C), suggesting that SOX9 is overexpressed in ESCC.

### SOX9 upregulation correlates with poor prognosis of human ESCC

**Figure 2:**
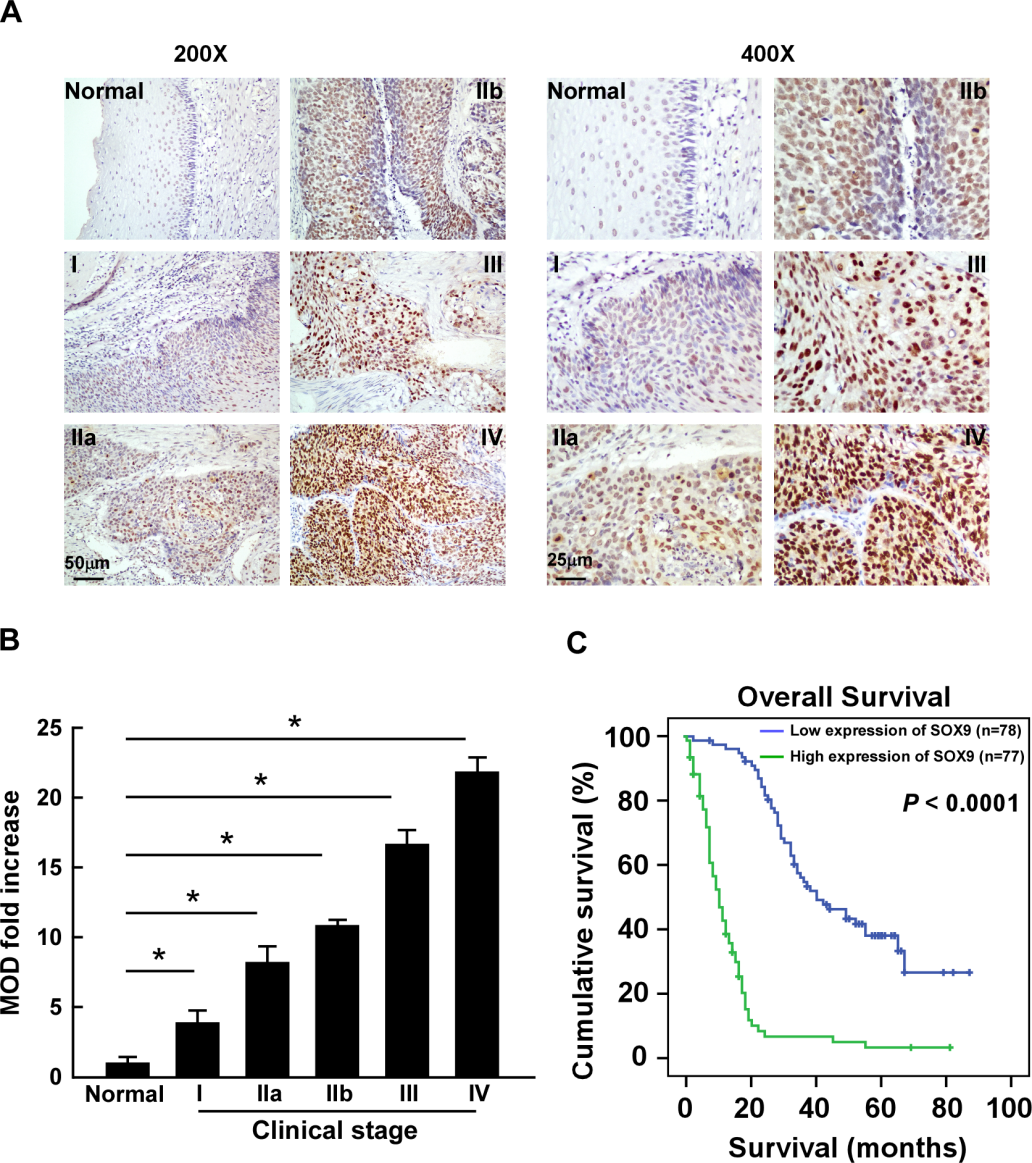
SOX9 upregulation correlates with poor prognosis of human ESCC **A.** Representative images of IHC analyses of ESCC specimens (clinical Stage I–IV tumors) compared with normal esophageal tissue (left, × 200 magnification; right, × 400 magnification). IHC analyses were performed in two independent experiments on sections from each specimen, with similar results. **B.** Statistical analyses of the mean optical density (MOD) of SOX9 staining between normal esophageal tissue and ESCC specimens from different clinical stages. Bars represent the mean ± SD of three independent experiments. *P < 0.05. **C.** Kaplan–Meier curves from univariate analyses (log-rank) of patients with low vs. high SOX9 expression. *P*-values were calculated using the log-rank test.

To determine the association between SOX9 expression and ESCC progression, the expression of SOX9 was examined by immunohistochemistry (IHC) in 155 paraffin-embedded, archived clinical ESCC specimens (Stage I, seven cases; Stage IIa, 70 cases; Stage IIb, 15 cases; Stage III, 56 cases; Stage IV, seven cases) (Supplementary Table 1). As shown in Figure 2A–2B, SOX9 was highly expressed in ESCC tissues of all stages as compared with the normal tissues. Furthermore, statistical analyses showed that SOX9 expression was positively correlated with tumor clinical stage (*P* < 0.001), T classification (*P* = 0.001), N classification (*P* < 0.001), M classification (*P* = 0.006), and pathological differentiation (*P* = 0.017) (Figure 2A and 2B; Table 1 and Supplementary Table 2). Moreover, SOX9 expression levels were inversely correlated with overall survival (*P* < 0.001; Figure 2C and Table 1), which was further confirmed by Spearman correlation analysis (*r* = −0.730, *P* < 0.001; Supplementary Table 2). Notably, SOX9 expression also correlated significantly with overall survival in both the Stage I + II (*n* = 77, *P* < 0.001) and Stage III + IV subgroups (*n* = 78, *P* < 0.001), as well as in the T1 + T2 (*n* = 47, *P* < 0.001) and T3 + T4 subgroups (*n* = 108, *P* < 0.001) (Supplementary Figure 2A–2B). In addition, univariate and multivariate analyses suggested that SOX9 expression was an independent prognostic factor in ESCC (Table 2).

**Table 1:** Correlation between SOX9 expression and clinicopathologic characteristics of ESCC

Characteristic		SOX9		Chi-square test *P*-value
		Low No. cases (%)	High No. cases (%)	
**Gender**	Male	54 (34.8)	62 (40.0)	0.105
	Female	24 (15.5)	15 (9.7)	
**Age (years)**	≦57	41 (26.5)	39 (25.1)	0.811
	>57	37 (23.9)	38 (24.5)	
**Clinical stage**	I	7 (4.5)	0 (0)	<0.001
	II	64 (41.3)	21 (13.6)	
	III	7 (4.5)	49 (31.6)	
	IV	0 (0)	7 (4.5)	
**T classification**	T1	8 (5.2)	1 (0.7)	0.001
	T2	27 (17.4)	11 (7.1)	
	T3	39 (25.2)	56 (36.1)	
	T4	4 (2.6)	9 (5.8)	
**N classification**	N0	67 (43.2)	18 (11.6)	<0.001
	N1	11 (7.1)	59 (38.1)	
**M classification**	M0	78 (50.3)	70 (45.2)	0.006
	M1	0 (0)	7 (4.5)	
**Differentiation**	Well	44 (28.4)	26 (16.8)	0.017
	Moderate	23 (14.8)	33 (21.3)	
	Poor	11 (7.1)	18 (11.6)	
**Survival status**	Alive	31 (20.0)	10 (6.5)	<0.001
	Death	47 (30.3)	67 (43.2)	

**Table 2:** Univariate and multivariate Cox regression analyses of prognostic parameters in patients with ESCC

	*Univariate analysis*		*Multivariate analysis*		
	*P*	Relative risk (SE)	*P*	Relative risk	95% confidence interval
**Gender**	0.019	0.583 (0.231)	0.073	0.651	0.408–1.041
**Age**	0.900	1.000 (0.001)	0.795	1.000	0.997–1.004
**Clinical stage**	<0.001	2.309 (0.101)	0.024	1.380	1.043–1.825
**T classification**	<0.001	1.869 (0.148)	0.245	1.244	0.861–1.797
**N classification**	<0.001	4.252 (0.209)	0.47	0.68	0.239–1.936
**M classification**	<0.001	7.049 (0.449)	0.050	2.688	0.999–7.234
**Differentiation**	<0.001	1.901 (0.126)	<0.001	1.689	1.292–2.208
**Tumor size**	<0.001	1.023 (0.004)	0.005	1.014	1.004–1.024
**Expression of SOX9**	<0.001	6.144 (0.21)	<0.001	3.699	2.049–6.677

### Overexpression of SOX9 promotes cell proliferation and tumorigenesis of ESCC *in vitro*

**Figure 3:**
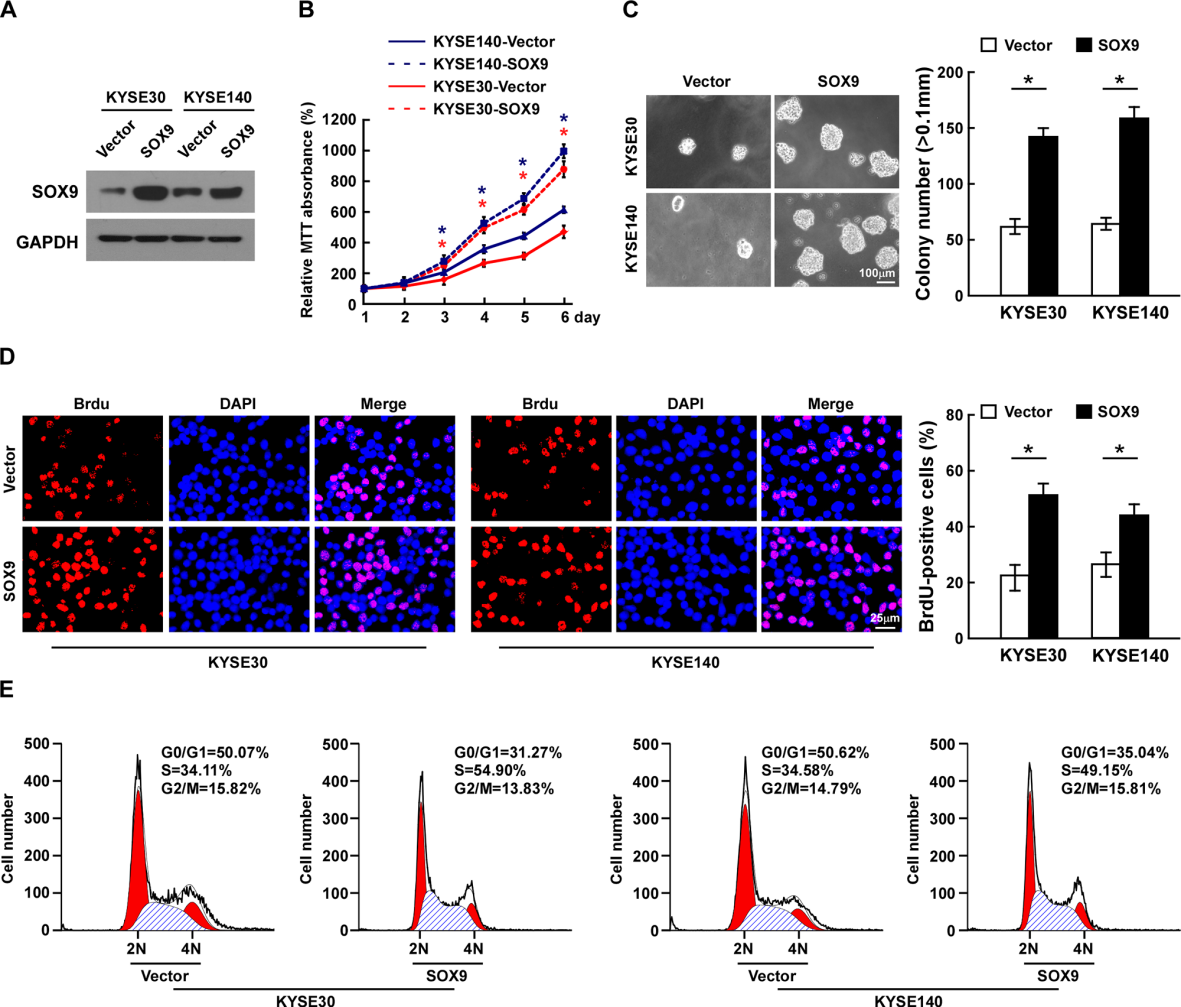
Overexpression of SOX9 promotes cell proliferation and tumorigenesis of ESCC *in vitro* **A.** Western blotting analysis of SOX9 expression in ESCC cell lines. **B.** Effects of SOX9 on cell proliferation as analyzed by MTT assay. **C.** Representative micrographs (left) and quantification (right) of colonies formed by cells as determined by anchorage-independent growth assay. **D.** Representative micrographs (left) and quantification (right) of BrdU incorporation assay. **E.** Cell cycle progression as measured by flow cytometry. All experiments were performed using KYSE30 and KYSE140 cells stably overexpressing SOX9 or pMSCV-vector. Bars represent the mean ± SD of three independent experiments. *P < 0.05.

As SOX9 expression was correlated with the clinical staging and T classification of ESCC (Table 2 and Supplementary Table 2), we further evaluated the effect of SOX9 on the tumorigenic activity of ESCC cells. KYSE30 and KYSE140 ESCC cell lines stably overexpressing SOX9 were established for further investigation (Figure 3A). The tetrazolium (MTT) assay showed that ectopic expression of SOX9 significantly increased the growth rate of the ESCC cells, and the anchorage-independent growth assay showed that ESCC cells stably overexpressing SOX9 formed more and larger colonies than the control cells (Figure 3B–3C). There were significantly more bromodeoxyuridine (BrdU)-positive cells among SOX9-transduced KYSE30 and KYSE140 cells, whereas the control cells displayed lower BrdU incorporation rates (Figure 3D). Moreover, flow cytometry revealed that, when SOX9 was overexpressed, there was a significant decrease in the percentage of cells in the G1/G0 phase and a dramatic increase in the percentage of cells in the S phase (Figure 3E). These results indicate that overexpression of SOX9 promotes the proliferation and tumorigenicity of ESCC cells *in vitro*.

### Silencing SOX9 decreases cell proliferation and tumorigenicity of ESCC

**Figure 4:**
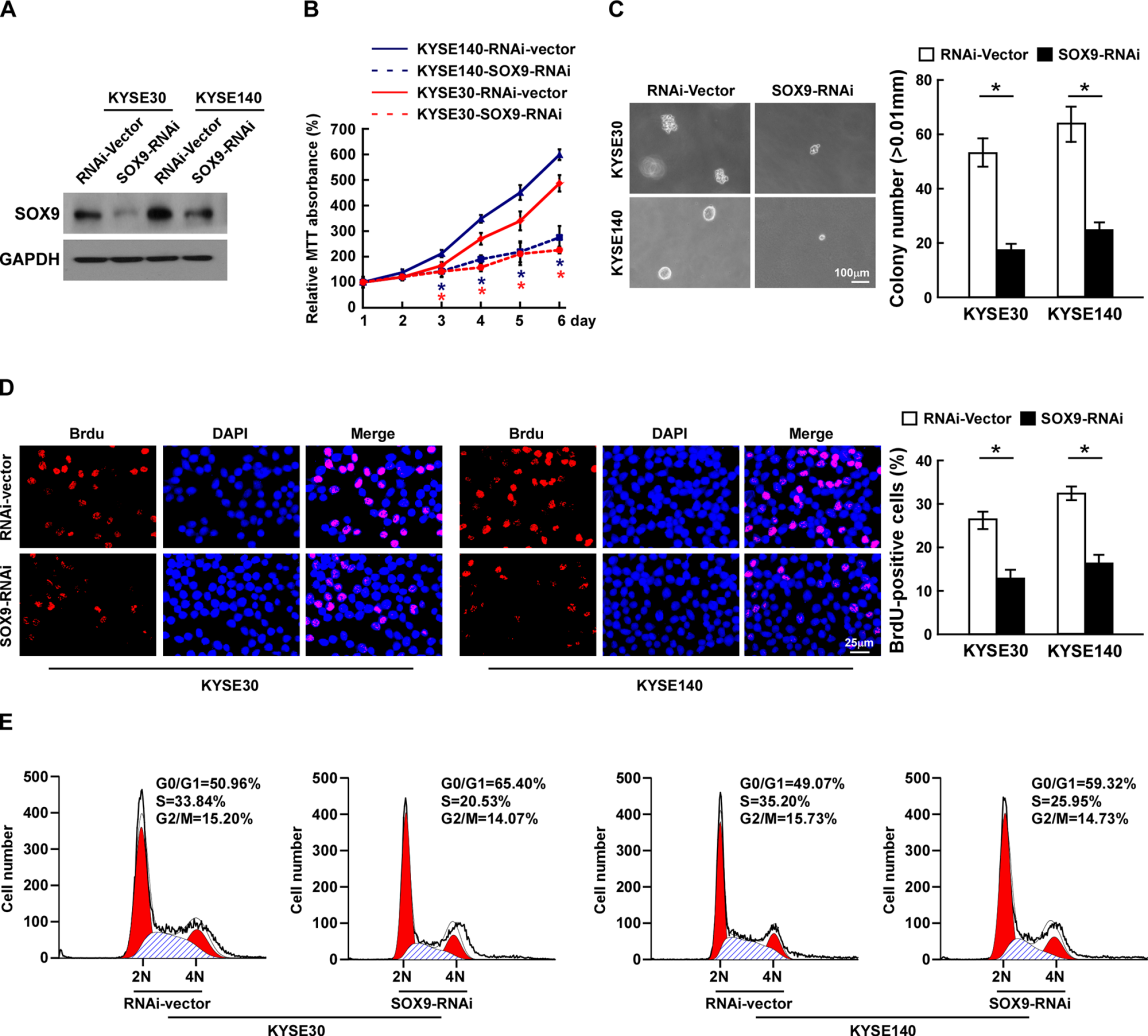
Silencing SOX9 suppresses the proliferation of ESCC cells **A.** Western blotting analysis of SOX9 expression in ESCC cell lines. **B.** Effects of SOX9 on cell proliferation as analyzed by MTT assay. **C.** Representative micrographs (left) and quantification (right) of colonies formed by cells as determined by anchorage-independent growth assay. **D.** Representative micrographs (left) and quantification (right) of the BrdU incorporation assay. **E.** Cell cycle progression as measured by flow cytometry. All experiments were performed using KYSE30 and KYSE140 cells in which SOX9 had been stably silenced or which expressed RNA interference (RNAi)-vector. Bars represent the mean ± SD of three independent experiments. *P < 0.05.

Loss-of-function studies using short hairpin RNA (shRNA) to silence SOX9 expression were performed to examine whether downregulation of SOX9 would inhibit ESCC cell proliferation (Figure 4A). As shown in Figure 4B–4D, silencing SOX9 drastically decreased the growth rate and anchorage-independent growth capability of ESCC cells. Furthermore, silencing SOX9 in ESCC cells dramatically decreased the percentages of BrdU-positive cells as compared to the control cells (Figure 4D). Moreover, flow cytometry revealed a significant increase in the percentage of cells in the G1/G0 phase and a decreased percentage of cells in the S phase among the SOX9-silenced cells as compared with the control cells (Figure 4E). These results indicate that downregulation of SOX9 suppresses the proliferation and tumorigenicity of ESCC cells *in vitro*.

### Upregulation of SOX9 enhances the tumorigenicity of ESCC cells *in vivo*

**Figure 5:**
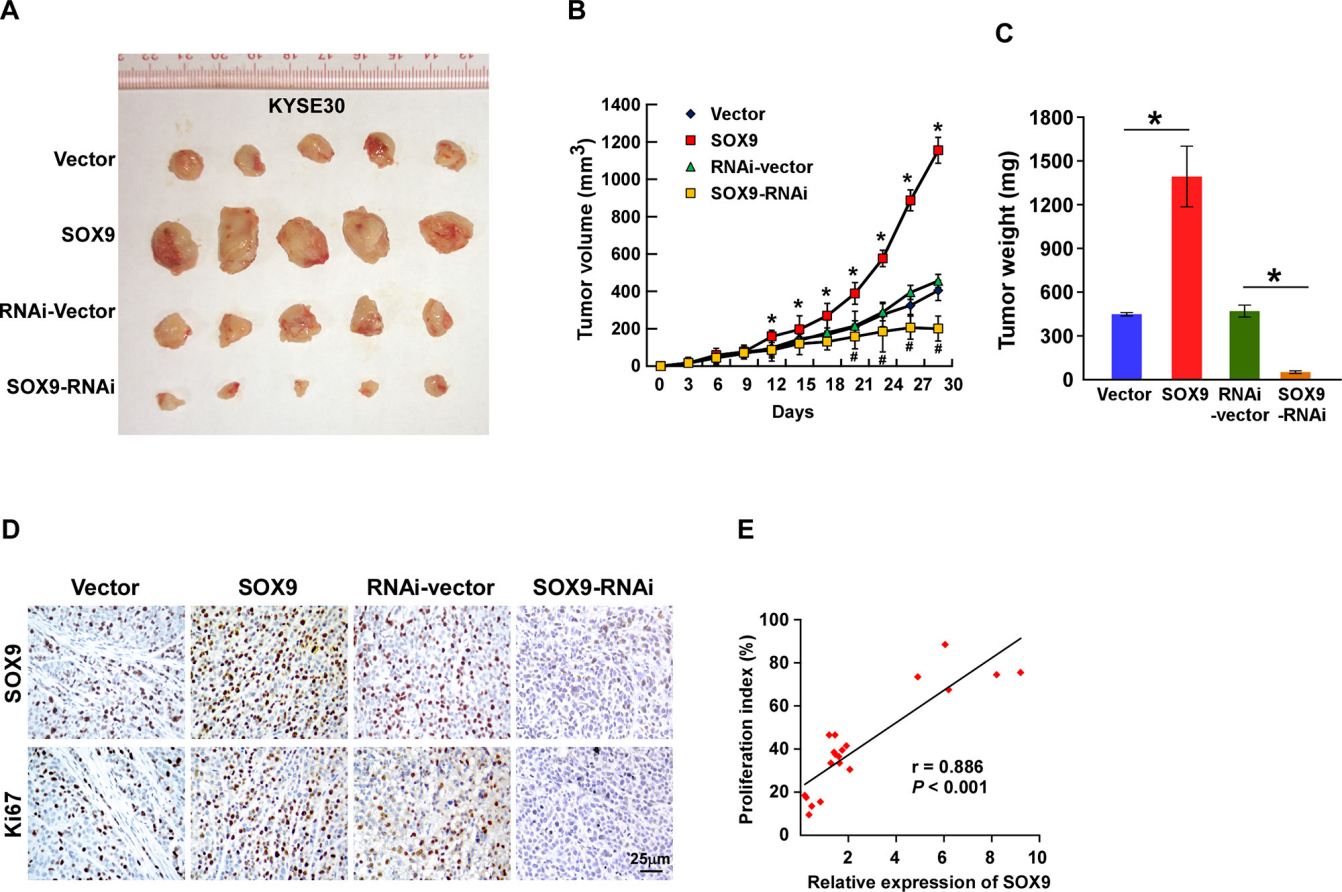
Upregulation of SOX9 enhances the tumorigenicity of ESCC cells *in vivo* **A–C.** Xenograft model in nude mice. KYSE30/Vector cells, KYSE30/SOX9 cells, KYSE30/RNAi-vector cells, or KYSE30/SOX9-RNAi cells were injected into the dorsal flanks of the mice (*n* = 5 per group). (A) Representative images of tumors from all mice in each group. (B) Mean tumor volume. (C) Mean tumor weights. **D.** IHC staining showing that overexpression of SOX9 induced the proliferation of ESCC cells *in vivo*, as indicated by the percentages of Ki67-positive cells, whereas downregulation of SOX9 inhibited it. **E.** Correlation between SOX9 expression and Ki67 as determined by IHC staining. Each bar represents the mean ± SD of three independent experiments. *P < 0.05.

The oncogenic role of SOX9 in ESCC progression was further examined using an *in vivo* tumor model. As shown in Figure 5A–5C, the tumors formed by SOX9-transduced ESCC cells were larger and heavier than the vector control tumors, whereas tumors formed by SOX9-silenced cells were smaller and lighter than the tumors formed by the control cells. Furthermore, IHC analysis showed that SOX9-overexpressing tumors had an increased Ki67 proliferation index, while SOX9-silenced tumors had fewer Ki67-positive cells (*r* = 0.886; *P* < 0.001; Figure 5D–5E and Supplementary Figure 3A–3C). Collectively, these results further support the premise that SOX9 contributes to ESCC proliferation.

### Overexpressing SOX9 activates the Akt signaling pathway

**Figure 6:**
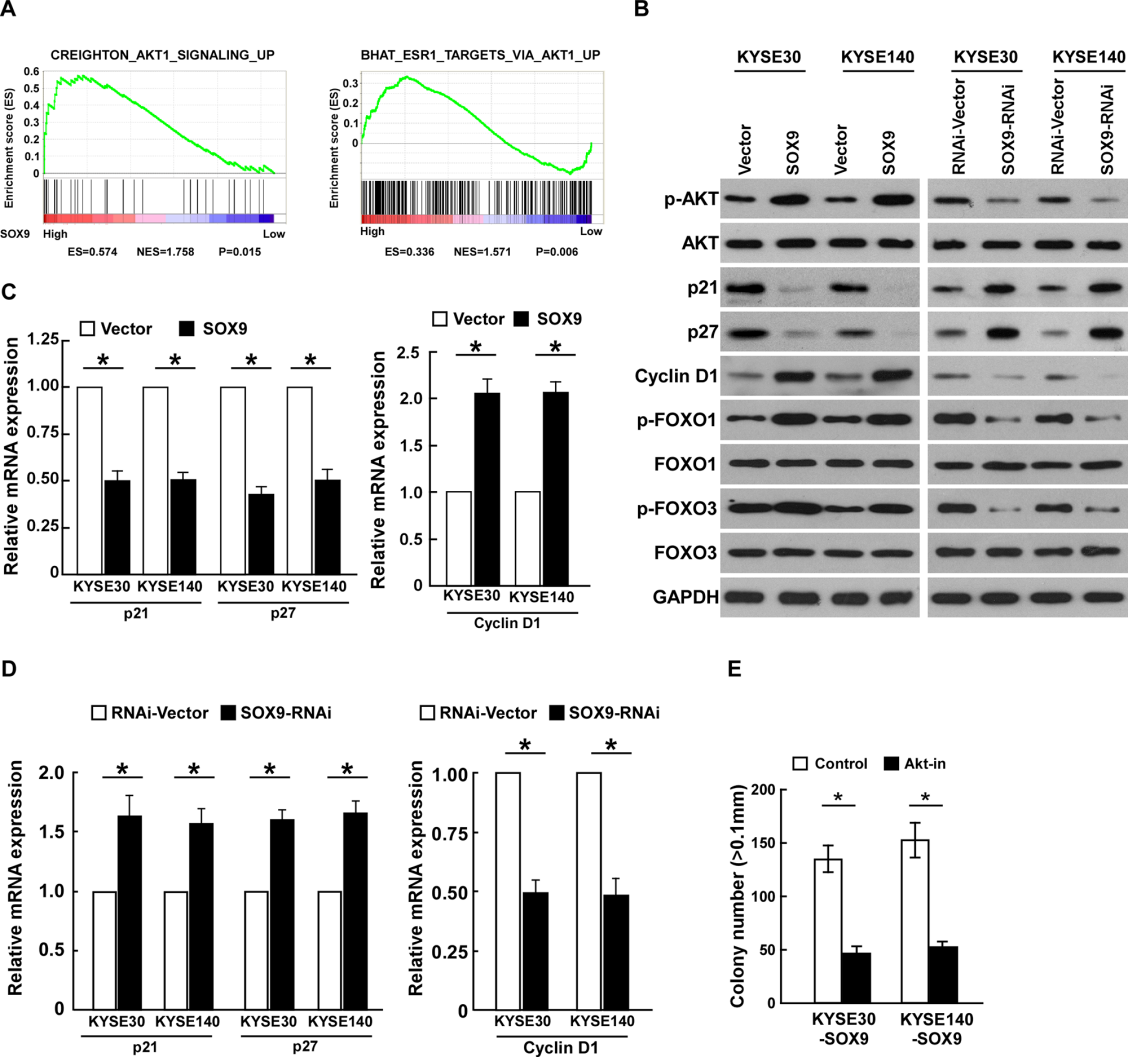
Overexpressing SOX9 activates the Akt signaling pathway **A.** GSEA analysis showing that SOX9 levels were significantly correlated with the Akt-downstream gene signatures (CREIGHTON___AKT1___SIGNALING___UP; BHAT___ESR1___ TARGETS___VIA___AKT1___ UP) in the TCGA database. **B.** Western blots of p-Akt, Akt, p21, p27, cyclin D1, p-FOXO1, total FOXO1, p-FOXO3, and total FOXO3 expression; GAPDH served as the loading control. **C–D.** Real-time PCR analysis of *P21*, *P27*, and cyclin D1 (*CCND1*) mRNA expression in ESCC cells. **E.** Quantification of colonies determined by anchorage-independent growth assay. Colonies larger than 0.1 mm in diameter were scored. Bars represent the mean ± SD of three independent experiments. *P < 0.05.

In an attempt to determine the underlying mechanism of SOX9 in ESCC progression, gene set enrichment analysis (GSEA) was performed using the TCGA database (http://cancergenome.nih.gov). As shown in Figure 6A, the level of SOX9 was significantly correlated with the Akt downstream gene signatures, suggesting that SOX9 might be involved in the activation of Akt signaling. Furthermore, the expression of p21^Cip1^ and p27^Kip1^, at both protein and mRNA level, was significantly downregulated in SOX9-overexpressing cells, but cyclin D1 expression was upregulated. Conversely, expression of p21^Cip1^ and p27^Kip1^ was increased in SOX9-downregulated cells, while that of cyclin D1 was decreased (Figure 6B–6D). Moreover, the expression of phosphorylated Akt (p-Akt), the upstream regulator of p21^Cip1^, p27^Kip1^, and cyclin D1, was dramatically elevated in SOX9-transduced cells but reduced in SOX9-silenced cells (Figure 6B). Overexpressing SOX9 dramatically increased the levels of phosphorylated forkhead box O (FOXO) 1 (p-FOXO1) and FOXO3 (p-FOXO3), the downstream factors of Akt (Figure 6B), but silencing SOX9 decreased them. Importantly, the stimulatory effect of SOX9 on the proliferation of ESCC cells was drastically reduced upon treatment with an Akt inhibitor (Figure 6E), demonstrating that functional Akt activation is vital to the biological effects of SOX9 in ESCC cells.

## DISCUSSION

In the present study, we provide evidence confirming the association between SOX9 expression and ESCC progression. SOX9 was upregulated at both mRNA and protein level in ESCC and SOX9 levels were correlated with poorer overall survival. Furthermore, we found that overexpression of SOX9 promoted the proliferation and tumorigenicity of ESCC cells both *in vitro* and *in vivo* but that knockdown of SOX9 inhibited it. Therefore, our results demonstrate that upregulation of SOX9 plays important roles in promoting ESCC progression.

ESCC, the most prevalent esophageal cancer subtype, has poor prognosis and a high mortality rate, and is more common in the developing countries, including China. It has been reported that China has both the highest ESCC morbidity and mortality worldwide, numbering 167,200 and 145,900 cases, respectively [3]. The high mortality of ESCC is mainly due to a lack of reliable methods for detecting the disease at the early stage [5]. Recently, numerous studies aimed to explore early predictive markers for early diagnosis of ESCC. Adenosine deaminase acting on RNA 1 (*ADAR1*), significantly overexpressed in ESCC, was reported as a potential biomarker of ESCC diagnosis and prognosis and as a new molecular therapeutic target [17]. Wang *et al*. demonstrated the association of Golgi phosphoprotein 3 (*GOLPH3*) with ESCC progression, cell differentiation, and survival, and concluded that *GOLPH3* was a possible diagnostic and prognostic marker of ESCC [18]. Other studies have found that microRNAs, such as miR-31, miR-183, miR-25, and miR-100, could also serve as useful biomarkers for diagnosing ESCC [19, 20]. Our study shows that SOX9 was upregulated in ESCC and was correlated with the malignant features of ESCC, such as clinical stage, tumor-nodes-metastasis (TNM) classification, tumor differentiation, tumor size, and survival, indicating that SOX9 could serve as a useful diagnostic and prognostic biomarker.

SOX9, a transcription factor essential for both sex and skeletal development, has been reported as an oncogenic gene or tumor suppressor, and it plays essential roles in various tumors. For example, SOX9 is upregulated in multiple malignancies, including prostate cancer, ovarian cancer, colorectal cancer, and hepatocellular carcinoma [12, 21–23]. SOX9 overexpression leads to the inhibition of T-cell factor (TCF)-binding activity and suppression of β-transducin repeat–containing protein (β-TrCP)-mediated protein degradation, promoting nuclear expression of glioma-associated oncogene homolog 1 (GLI1), resulting in enhancement of cancer stem cell properties [24]. However, paradoxical studies about SOX9 expression and function in tumors remain. Overexpression of SOX9 inhibits the growth and tumorigenicity of prostate cancer cells, attenuates melanoma cell growth, and restores sensitivity to retinoic acid treatments [25, 26]. Jay *et al*. found that upregulation of SOX9 repressed the expression of the human carcinoembryonic antigen (*CEA*) gene, which is overexpressed in many tumors and could be used as a clinical biomarker for tumor diagnosis, and consequently led to apoptosis of colon cancer cells [27]. However, the expression and function of SOX9 in ESCC has not been elucidated.

Barrett's esophagus (BE) can evolve to esophageal adenocarcinoma (EAC) through low-grade dysplasia and high-grade dysplasia (HGD), and SOX9 expression is significantly increased in the upper crypt epithelial cells in HGD [28]. Furthermore, Clemons and colleagues demonstrated that SOX9 is sufficient for driving columnar differentiation of squamous epithelium and the expression of an intestinal differentiation marker, reminiscent of BE [29]. These results suggest that SOX9 may be an important early event in the development of BE and/or EAC. In our study, SOX9 was also markedly expressed in early-stage ESCC tissues (Stage I) as compared with normal tissues, suggesting that upregulation of SOX9 might be involved in the initiation of ESCC. Therefore, investigating the clinical significance of SOX9 in esophageal dysplasia and the biological role of SOX9 upregulation in the proliferation of normal squamous epithelium further would be worthwhile, as it might represent a novel predictor of ESCC.

The phosphoinositide 3-kinase (PI3K)/Akt pathway is a major signaling cascade that is activated in a large variety of human cancers [30]. Our study demonstrates that SOX9 dramatically activated the PI3K/Akt signaling pathway, followed by the upregulation of p-Akt, cyclin D1, p-FOXO1, and p-FOXO3, and the downregulation of p21^Cip1^ and p27^Kip1^, resulting in the promotion of ESCC proliferation and tumorigenicity. As activation of the PI3K/Akt pathway also induces a series of genes involved in metastasis, angiogenesis, or other malignant characteristics [31, 32], it would be interesting to investigate the potential function of SOX9 in ESCC angiogenesis or other neoplastic function further. SOX9 is correlated with multiple malignant characteristics and exhibits several pro-oncogenic properties, including the ability to promote proliferation, inhibit senescence, and collaborate with other oncogenes in malignant transformation [33]. Furthermore, knockdown of SOX9 expression reduces the invasiveness and metastasis of colon cancer cells [34]. Liu *et al*. found that overexpression of SOX9 in U251 glioma cells significantly increased cell migration and invasion [35]. Capaccione and colleagues found that SOX9 participates in Notch-1–induced lung cancer cell motility, cell invasion, and loss of E-cadherin expression [14]. These results indicate that SOX9 is involved in cancer invasion and metastasis. Consistently, we found that SOX9 expression was positively correlated with N classification (*P* < 0.001) and M classification (*P* = 0.006) (Supplemental Table 2 and 3), suggesting that SOX9 might play roles in ESCC cell invasion or metastasis. Collectively, the function of SOX9 in tumors is paradoxical and complicated, suggesting a corresponding complex regulatory mechanism.

The cause of SOX9 deregulation in cancer is another issue of interest. Cai *et al*. reported that SOX9 is a major downstream effector of avian v-ets erythroblastosis virus E26 oncogene homolog (ERG) in prostate cancer, resulting in neoplasia in murine prostate and in the stimulation of tumor growth and invasion [36]. In esophageal cancer, the Hippo coactivator Yes-associated protein 1 (YAP1) upregulates SOX9 transcription by binding to the *SOX9* promoter, upregulating SOX9 expression, followed by the acquisition of cancer stem cell properties [37]. Via ECR Browser analysis, we also found multiple potential transcriptive binding sites for various transcriptors, including FOXOs, signal transducer and activator of transcription proteins (STATs), activation protein-1 (AP1), and GATA. Collectively, the mechanism of SOX9 upregulation in ESCC warrants further exploration, and it might be helpful for studying SOX9 network regulation.

## MATERIAL AND METHODS

### Cell lines and culture

Primary cultures of normal esophageal epithelial cells (NEEC) were established from fresh specimens of the adjacent noncancerous esophageal tissue, which is over 5 cm from the cancerous tissue, according to previous report [38]. The ESCC cell lines, purchased from Bogoo Co. (Shanghai, China), including EC18, KYSE30, KYSE140, KYSE180, KYSE410, KYSE510, KYSE 520, 108Ca, TE-1, ECa109, EC18 and HKESC1 were grown in DMEM medium (Invitrogen, Carlsbad, CA, USA) supplemented with 10% fetal bovine serum (Invitrogen) and 100 μg/ml penicillin, and 100 μg/ml streptomycin (Invitrogen) at 37°C in a humidified atmosphere containing 5% CO_2_.

### Patient information and tissue specimens

The current study was conducted using eight pairs of matched ESCC tissues and the adjacent non-cancerous tissues and 155 paraffin-embedded samples histopathologically and clinically diagnosed with ESCC from 2001 to 2006 at the Shenzhen People's Hospital in patients who had undergone esophageal cancer resection prior to the administration of chemotherapy (Supplementary Table 1). The ESCC and carcinoma-adjacent samples were obtained from resected tumors and the adjacent non-tumor esophageal tissues, respectively, and confirmed by pathological review. All samples were obtained from the Shenzhen People's Hospital Tissue Bank and coded anonymously in accordance with local ethical guidelines. We obtained approval from the Shenzhen People's Hospital Review Board and obtained informed patient consent in accordance with the Declaration of Helsinki. ESCC specimens were staged in accordance with American Joint Cancer Committee/Union Internationale Contre le Cancer classification guidelines. ESCC specimen grading and histopathology subtyping were based on World Health Organization criteria. The eight ESCC biopsy samples and their matched adjacent noncancerous esophageal tissues were frozen in liquid nitrogen and stored.

### RNA extraction and real-time reverse transcription (RT)-PCR

Total RNA from cells and tissues was extracted using TRIzol reagent (Invitrogen Life Technologies, Ontario, Canada). The qPCR primers for amplifying SOX9 were designed using the qPrimerDepot website (http://primerdepot.nci.nih.gov/). The SOX9 primers were: forward, 5′-GTGGTCCTTCTTGTGCTGC-3′ and reverse, 5′-GTACCCGCACTTGCACAA C-3′; the glyceraldehyde 3-phosphate dehydrogenase (GAPDH) primers were: forward, 5′-ATTCCACCCATGGCAAATTC-3′ and reverse, 5′-ATTCCACCCATGGCAAATTC-3′. The qRT-PCR was carried out using FastStart Universal SYBR Green Master (ROX; Roche, Toronto, Canada) on a Bio-Rad CFX96 qRT-PCR detection system (Bio-Rad Laboratories, Hercules, CA, USA). For PCR amplification of cDNA, an initial amplification using specific primers was done with a denaturation step at 94°C for 10 min, followed by 30 cycles of denaturation at 94°C for 45 s, primer annealing at 58°C for 30 s, and primer extension at 72°C for 25s. Upon completion of the cycling steps, a final extension at 72°C for 6 minutes was done before the reaction was stored at 4°C. CFX Manager software (Bio-Rad Laboratories) was used to calculate the threshold cycle (Ct) value for GAPDH and SOX9 during the log phase of each cycle. Expression data were normalized to the geometric mean of housekeeping gene GAPDH to control the variability in expression levels and calculated as 2^−[(C t of SOX9) – (C t of GAPDH)]^, where Ct represents the threshold cycle for each transcript. To minimize experimental variability, each sample was tested in triplicate and the mean femtogram expression level was calculated.

### Plasmids and retroviral infection

A SOX9 expression construct was generated by subcloning PCR-amplified full-length human SOX9 cDNA into the pMSCV vector. For depletion of SOX9 to silence endogenous SOX9, a short hairpin RNA (shRNA) oligonucleotides were cloned into the pSuper-retro-puro vector to generate pSuper-retro-SOX9-RNAi. Stable cell lines expressing SOX9 and SOX9-shRNA were generated via retroviral infection using HEK293T cells as previously described [39] and selected with puromycin (0.5 μg/ml) 48 h after infection, for 10 days.

### Western blotting analysis

Total protein was extracted from whole cells and 20 μg isolated protein was separated by sodium dodecyl sulfate–polyacrylamide gel electrophoresis and electroblotted onto a polyvinylidene fluoride membrane (Bio-Rad Laboratories, Hercules, CA, USA). The membrane was probed with anti-SOX9 (56KD) mouse monoclonal antibody (1:500; #ab76997, Abcam, Cambridge, MA, USA), anti-p-Akt(#4056), anti-Akt(#9272), anti-p21(#2947), anti-p27(#3686), anti-CyclinD1(#2922), anti-p-FOXO1(#9461), anti-FOXO1(#9454), anti-p-FOXO3(#9465) or anti-FOXO3(#2472) (Cell Signaling, Danvers, MA, USA). The membranes were then stripped and reprobed with an anti–α-tubulin mouse monoclonal antibody (1:1000; #2125; Cell Signaling, Danvers, MA, USA) as the loading control. Bound antibodies were visualized using an enhanced chemiluminescence system (Amersham Pharmacia Biotech, Dübendorf, Switzerland).

### Immunohistochemistry (IHC)

Formalin-fixed paraffin-embedded tissues were analyzed using immunohistochemical staining as previously described [21], using anti-SOX9 antibody (#ab76997, Abcam) with a dilution rate of 1:50. Two independent pathologists uninformed of the histopathological features and patient data of the samples separately reviewed and scored the degree of immunostaining of the sections. Scores were determined by combining the proportion of positively stained tumor cells and the staining intensity. The scores assigned independently by the two pathologists were combined into a mean score for further comparative evaluation. Tumor cell proportions were scored as follows: 0 (no positive tumor cells); 1 (< 10% positive tumor cells); 2 (10–35% positive tumor cells); 3 (35–75% positive tumor cells), or 4 (> 75% positive tumor cells). Staining intensity was graded as follows: 1 (no staining); 2 (weak staining = light yellow); 3 (moderate staining = yellow brown), or 4 (strong staining = brown). The staining index (SI) was calculated as the product of the staining intensity score and the proportion of positive tumor cells. Using this assessment method, SOX9 expression in ESCC was evaluated using the SI (scored as 0, 1, 2, 3, 4, 6, 8, 9, or 12). A SI score ≥ 6 indicated high expression, while scores < 6 indicated low expression.

IHC staining of the tumor and normal tissues was analyzed quantitatively using an AxioVision Rel. 4.6 computerized image analysis system (Carl Zeiss, Oberkochen, Germany). Stained sections were evaluated at × 200 magnification; 10 representative fields from each section were analyzed to obtain the mean optical density (MOD), which represents the strength of staining signals as measured per positive pixels [40–42]. In brief, the stained slides were evaluated at 200x magnification using the SAMBA 4000 computerized image analysis system with Immuno 4.0 quantitative program (Image Products International, Chantilly, Virginia). Ten representative staining fields of each tumor sample were analyzed to determine the Mean Optical Density (MOD), which represents the concentration of the stain as measured per positive pixels in the whole tissue. A negative control with each batch of staining was used for background subtraction in the quantitative analysis. The MOD data were statistically analyzed using *t*-test to compare the average MOD difference between different group of tissues, *P* < 0.05 was considered significant.

### 3-(4, 5-Dimethyl-2-thiazolyl)-2, 5-diphenyl- 2H-tetrazolium bromide (MTT) assay

Cells were seeded in 96-well plates at initial density of (0.2 × 10^4^/well). At each time point, cells were stained with 100 μl sterile MTT dye (0.5 mg/ml, Sigma) for 4 h at 37°C, followed by removal of the culture medium and addition of 150 μl of dimethyl sulphoxide (DMSO) (Sigma, St. Louis, MO, USA). The absorbance was measured at 570 nm, with 655 nm as the reference wavelength. All experiments were performed in triplicates.

### Anchorage-independent growth ability assay

Five hundred cells were trypsinized and suspended in 2 ml complete medium plus 0.3% agar (Sigma, Saint Louis, MI). The agar-cell mixture was plated on top of a bottom layer with 1% agar completed medium mixture. At 10 days, viable colonies larger than 0.1 mm were counted. The experiment was performed for three independently times for each cell line.

### Bromodeoxyuridine labeling and immunofluorescence

Cells were plated on coverslips (Fisher, Pittsburgh, PA). After 24 hours, cells were incubated with bromodeoxyuridine (BrdUrd) for 1 h and stained with anti-BrdUrd antibody (Upstate, Temecula, CA, USA) according to the manufacturer's instruction. Gray level images were acquired under a laser scanning microscope (Axioskop 2 plus, Carl Zeiss Co. Ltd., Jena, Germany).

### Flow cytometry analysis

Cells were harvested by trypsinization, washed with PBS, and fixed in 80% ice-cold ethanol in PBS. The cells were then pelleted and resuspended in cold PBS. Bovine pancreatic RNAase (2 μg/ml, Invitrogen) was added and cells were incubated at 37°C for 30 min. Propidium iodide (10 μg/ml, Invitrogen) was added and incubated for 30 min at room temperature. 3 × 10^4^ cells were analyzed by flow cytometry (FACSCalibur; BD Biosciences, San Jose, CA, USA). All experiments were performed in triplicates.

### Cell Treatments

Akt Inhibitor MK-2206 (1 μM; Selleck Chemicals, Houston, TX, USA) was dissolved in dimethyl sulfoxide. Single-cell suspension at a density of 500 cells per well was seeded in six-well plates. On the second day, cells were treated with the Akt inhibitor MK-2206 (1.0 μM). The medium was replaced with fresh medium containing MK-2206 every 3 days. At 10 days, viable colonies that were larger than 0.1 mm were counted. The experiment was performed for three independently times for each cell line.

### Xenografted tumor model

Female BALB/c-nu mice (4–5 weeks of age, 18–20g) were purchased from the Center of Experimental Animal of Guangzhou University of Chinese Medicine. All experimental procedures were approved by the Institutional Animal Care and Use Committee of Southern Medical University. The BALB/c nude mice were randomly divided into two groups (*n* = 5/group). One group of mice was inoculated subcutaneously with KYSE30/Vector cells (5 × 10^6^) in the left dorsal flank and with KYSE30/SOX9 cells (5 × 10^6^) in the right dorsal flank per mouse. Another group was inoculated subcutaneously per mouse with KYSE30/RNAi Vector cells (5 × 10^6^) in the left dorsal flank and with KYSE30/SOX9 RNAi cells (5 × 10^6^) in the right dorsal flank. Tumors were examined twice weekly; length, width, and thickness measurements were obtained with calipers and tumor volumes were calculated. Tumor volume was calculated using the equation (L * W^2^)/2. On day 27, tumors were detected by an IVIS imagining system (Caliper), then animals were euthanized, tumors were excised, weighed and paraffin-embedded. Serial 6.0 μm sections were cut and subjected to IHC and H&E staining. After deparaffinization, sections were IHC analyzed using an anti-Ki67 antibody (Dako, Denmark, Glostrup) or H&E stained with Mayer's hematoxylin solution. The images were captured using the AxioVision Rel.4.6 computerized image analysis system (Carl Zeiss). Proliferation index was quantized by counting proportion of Ki67-positive cells.

### Statistical analysis

The relationship between SOX9 expression and patient age, sex, tumor stage, TNM classification, histological differentiation, tumor size, degree of invasion, and patient overall survival was studied using Pearson's chi-square test and Spearman correlation analysis. Survival curves for patients with high and low SOX9 expression were plotted using the Kaplan–Meier method; statistical differences were compared using log-rank testing. Univariate and multivariable survival analysis was performed using Cox regression analysis. *P* < 0.05 was considered statistically significant. All statistical analyses were performed using SPSS 13.0 (SPSS Inc., Chicago, IL, USA).

## SUPPLEMENTARY FIGURES AND TABLES


