# Supplementary material for: Upregulation of sex-determining region Y-box 9 (SOX9) promotes cell proliferation and tumorigenicity in esophageal squamous cell carcinoma
Source: Oncotarget. 2015 Sep 4;6(31):31241–54. doi: 10.18632/oncotarget.5160 (PMC4741601; doi:10.18632/oncotarget.5160)
Supplement: Supplementary file 1 [file oncotarget-06-31241-s001.pdf]

SUPPLEMENTARY FIGURES AND TABLES

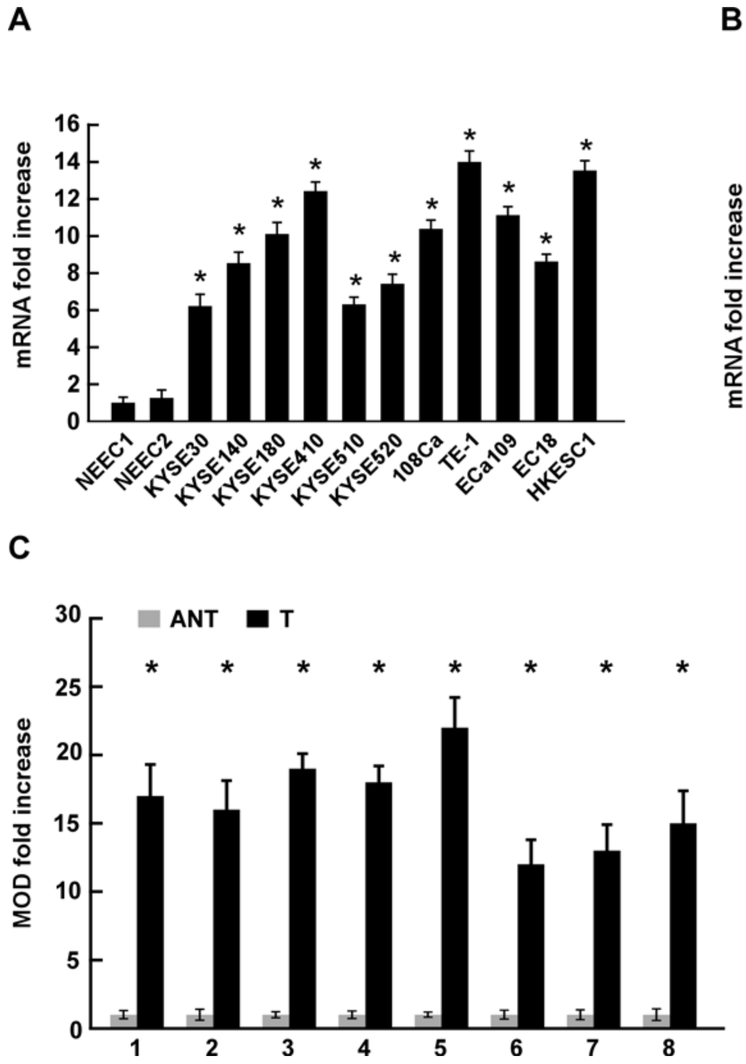

Supplementary Figure S1: SOX9 is overexpressed in ESCC cell lines and ESCC tissues. A–B. Real-time PCR analysis of *SOX9* expression in ESCC cell lines (A) and in eight paired ESCC (T) and adjacent non-tumor tissue (ANT) specimens (B). **C.** Statistical analyses of the MOD of SOX9 staining in eight paired ESCC (T) and adjacent non-tumor tissue (ANT) specimens. Bars represent the mean ± SD of three independent experiments. *$P < 0.05$.

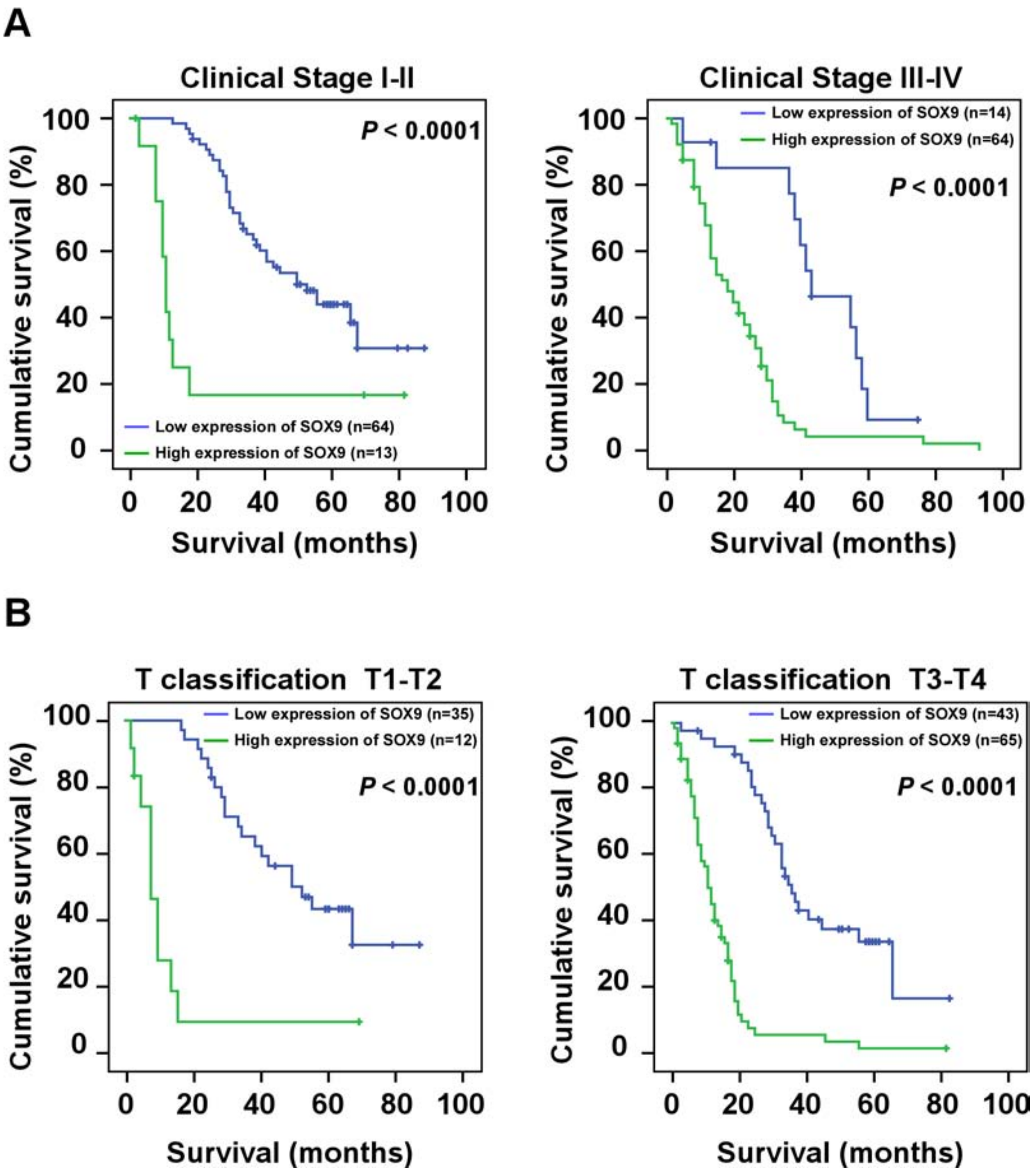

Supplementary Figure S2: Kaplan–Meier analysis of correlation between SOX9 protein levels and survival in patient subgroups. A. Comparison of the statistical significance of the difference between the survival curves of patients with high and low SOX9 expression in the clinical Stage I+II (left) or clinical Stage III+IV (right) subgroups. **B.** Comparison of the statistical significance of the difference between the survival curves of patients with high and low SOX9 expression in the T1–T2 (left) and T3–T4 (right) subgroups. *P*-values were calculated using the log-rank test.

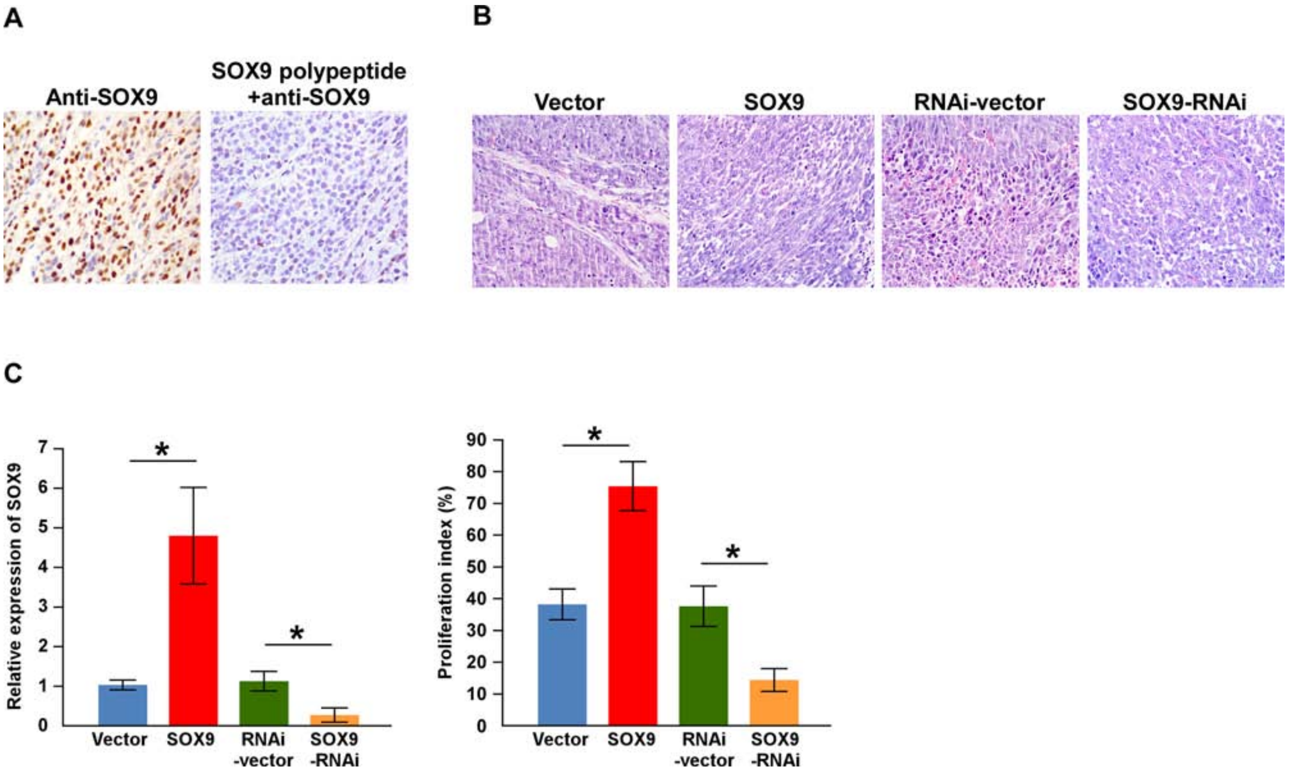

Supplementary Figure S3: Upregulation of SOX9 enhances the tumorigenicity of ESCC cells *in vivo*. A. The specificity of anti-SOX9 antibody was verified by IHC staining with anti-SOX9 or anti-SOX9 combined with recombinant SOX9 polypeptide, which completely blocked the staining by anti-SOX9. **B.** Hematoxylin and eosin staining of the tumors in the mice. **C.** Relative expression of SOX9 and the proliferation index indicating Ki67 expression as determined by IHC staining in the mice.

Supplementary Table S1: Clinicopathological characteristics of patient samples and SOX9 expression in ESCC

	Number of cases (%)
**Gender**	
Male	116(74.8)
Female	39(25.2)
**Age (years)**	
≦57	80(51.6)
>57	75(48.4)
**Clinical stage**	
I	7(4.5)
IIA	70(45.2)
IIB	15(9.7)
III	56(36.1)
IV	7(4.5)
**T classification**	
T1	9(5.8)
T2	38(24.5)
T3	95(61.3)
T4	13(8.4)
**N classification**	
N0	85(54.8)
N1	70(45.2)
**M classification**	
M0	148(95.5)
M1	7(4.5)
**Differentiation**	
Well	70(45.2)
Moderate	56(36.1)
Poor	29(18.7)
**Vital status (at follow-up)**	
Alive	41(26.5)
Death	114(73.5)
**Expression of SBSN**	
Low expression	78(50.3)
High expression	77(49.7)

Supplementary Table S2: Spearman correlation analysis of SOX9 expression and clinical pathologic factors

Variables	SOX9 expression level	
	Spearman correlation	*P*-value
**Gender**	−0.130	0.107
**Age**	0.026	0.745
**Clinical stage**	0.683	<0.0001
**T classification**	0.323	<0.0001
**N classification**	0.628	<0.0001
**M classification**	0.219	0.006
**Differentiation**	0.220	0.006
**Tumor size**	0.367	<0.0001
**Survival time**	−0.730	<0.0001
**Survival status (Alive/death)**	0.303	<0.0001
